# Predicting Geolocation of Tweets: Using Combination of CNN and BiLSTM

**DOI:** 10.1007/s41019-021-00165-1

**Published:** 2021-07-08

**Authors:** Rhea Mahajan, Vibhakar Mansotra

**Affiliations:** Department of Computer Science and IT, University of Jammu, Jammu, J&K India

**Keywords:** Twitter, Social networking platform, Bidirectional long short-term memory, Geolocation, Convolutional neural network

## Abstract

Twitter is one of the most popular micro-blogging and social networking platforms where users post their opinions, preferences, activities, thoughts, views, etc., in form of tweets within the limit of 280 characters. In order to study and analyse the social behavior and activities of a user across a region, it becomes necessary to identify the location of the tweet. This paper aims to predict geolocation of real-time tweets at the city level collected for a period of 30 days by using a combination of convolutional neural network and a bidirectional long short-term memory by extracting features within the tweets and features associated with the tweets. We have also compared our results with previous baseline models and the findings of our experiment show a significant improvement over baselines methods achieving an accuracy of 92.6 with a median error of 22.4 km at city level prediction.

## Introduction

Social Networking platforms not only play a prominent role in connecting people all over the world but they also have the hidden potential to uncover interesting patterns and significant bits of knowledge when a factual examination is applied to their unstructured data. The huge and tremendous utilization of these sites which collects massive amount of data on our area, activities, interests and preferences provide unparallel opportunities to track the movement of  its users. A study into this pattern of human movement, in light of the information from our versatile applications, frequently shows how predictable a considerable lot of our activities are; as user behavior on social media is an image of their actions and activities in actual life [1]. Social Media data which comes under the domain of Big Data is enormously large data that is growing at an unprecedented rate. Every second, on average, around 7000 tweets are posted on Twitter, which corresponds to over 400,000 tweets sent per min, 500 million per day and around 250 billion tweets per year [2].

With this huge and unparalleled rate of content generation, individuals are easily overwhelmed with data but find it difficult to discover content that is relevant to their interests. So, extracting actionable patterns of the user behavior, their movement across a region and trends from Twitter data can be called Tweet mining.

Twitter allows its users to share their geolocation with the facility of GPS function yet less than 1% of the users choose to conceal their geo-location in order to maintain privacy or prevent bullying, stalking or trolling [3]. Geographic location information of social media users can also provide great assistance and insights in crime prediction and prevention such as cyberstalking, cyberbullying or suicide if a user is exhibiting suspicious behavior in his/her Tweet [4]. Knowing the location of social media users is also important for location-specific services and recommendations, earth quake relief detection, natural disaster management [5], demographic analysis and health care management [6] especially in the time of  the COVID-19 pandemic [7].

In this paper, we have proposed a model to solve the problem of geolocation prediction of Tweets by combining two neural networks, CNN and BiLSTM. The intention of combination of these two deep learning techniques is to take the benefit of the advantages of CNN and BiLSTM architecture.

While CNN has the ability to utilize its structure of multi-layer perceptron to extract high level features in the text and has a decent capability to absorb complex, and non-linear mapping relationship from text. LSTMs generally take advantage of their ability to capture long-term dependencies between the text. We preferred to use BiLSTM instead of RNN and LSTM as BiLSTM is known to solve the problem of gradient disappearance or explosion which may occur in RNN. Moreover, BiLSTM provides additional training by scanning the data two times, from left to right and, right to left thus, extracting the semantics of a word in the context of the information preceding and succeeding it. The strength of our proposed technique is that it enables extracting the maximum amount of information from the data using convolutional layers while maintaining the chronological order between the data by traversing it in both directions using BiLSTM [8].

This paper is organized as follows: after introduction in Sect. 1, Sect. 2 provides an outline of related works for location prediction of tweets. In Sect. 3, we describe the data set used and the architecture of  the proposed model is elaborated in Sect. 4. Theoretical analysis of the model in terms of time and space complexity is stated in Sect. 5. Results obtained by performing experiments on the testing data on different evaluation metrics are presented in Sects. 5 and 6. Finally in Sect. 7, we have concluded the paper with a comparison of our model to previous baseline models and some potential future work.

## Related Works

Due to the lack of geotagged tweets and untrustworthiness of user declared location on Twitter, there is growing interest in researchers in predicting tweet location. Earlier studies on geolocation prediction of tweets mostly used machine learning techniques [9]. Han et al. (2012) applied Naïve bayes and Logistic Regression to find location of the tweets by extracting location indicative words and hashtags in the tweets. A year later, they proposed a stacking-based approach [10] that used a combination of tweet content and metadata to improve their results. Further, Han et al. [11] assessed the impact of non-geotagged tweets, language, and user-declared metadata on geolocation prediction and deliberated how user behavior can differ in terms of their location or region. However, these approaches didn’t fit well with the enormous volume of data available on Twitter.

Recent studies have shifted the paradigm from machine learning techniques to deep learning approaches for location prediction of Twitter users. Huang and Carley [12] integrated tweet text and user profile meta data in one model using convolutional neural network. Their proposed model showed better accuracy but their results were partial because data was highly skewed toward few cities. Further Huang and Carley [13] presented a hierarchical location prediction neural network (HLPNN) which incorporated network features apart from tweet text and associated meta data. Though their model was flexible in accommodating different feature combinations but ignored dynamic user movement. Huang et al. [14] introduced a multi-head self-attention model for text representation with sub word feature and CNN to improve the accuracy but ignored the semantics to capture the meaning of the tweet. Table 1 lists summary of the earlier works in the area of geolocation prediction of tweets.

**Table 1:** Chronologically lists some of the important works in geolocation prediction of tweets

	Data set	Features used	Techniques
Han et al. [9]	The regional North America geolocation Dataset, WORLD	Location indicative words	Naïve Bayes and Logistic regression
Han et al. [10]	WORLD	Tweet text and Meta data	Topic-based modeling using mutinomial Naïve Bayes classifier
Han et al. [11]	WORLD	Location indicative words, hashtags, user mentions and meta data	Naïve Bayes and logistic regression
Huang and Carley [12]	Real time tweets	Tweet text and meta data	CNN
Huang and Carley [13]	Twitter US, Twitter World, W-NNUT	Tweet text, meta data and network features	Hierarchical Method using neural network
Huang et al. [14]	W-NUT 2016	Subword feature	Multihead self- attention mechanism and CNN
Our approach	Real time geo-tagged English language Tweets collected across 10 cities of India	Tweet text, user self declared home location, and User display name in word embedding	Combination of CNN and BiLSTM

In our proposed study, we have tried to overcome the above limitations by collecting real-time tweets across 10 cities of India to find from where the tweet has been posted rather than using already available Data sets. Moreover, we have developed our training set that is evenly distributed across the cities. In our study, emphasis has been laid on geo-location prediction of tweet at the city level and the results presented clearly indicate predicted output probability of the tweets coming from each city which is lacking in studies of earlier researchers. Further, we have pre-processed our tweets to remove any noise using Natural language Processing. Lastly, we have combined two deep learning techniques which makes our model more robust and outperforms previous baseline models in terms of accuracy. Moreover, deep learning-based algorithms have shown to offers better predictions results as compared to machine learning algorithms on Big Data analytics.

## Dataset Description

To extract Twitter data, we must first create a Twitter account. Then, Twitter needs its users to sign up for an application. This application verifies our account and provides the user with an access token and consumer key, which can subsequently be used to connect to Twitter and retrieve tweets. The Twitter streaming API was used to gather real-time geo-tagged tweets across 10 cities of India for a period of 30 days from 1 August 2020 to 30 August 2020. Using Google’s geo-coding API,^1^ first we obtained a bounding box in terms of latitude and longitude for each city. Then, the geo-tag filter option of Twitter’s streaming API was used to extract tweets for each of those bounding boxes until we received 45,678 tweets from 21,544 unique users (Table 2).

**Table 2:** Dataset description

No. of tweets	No. of users	Country	Cities	Time zone
45,678	21,544	India	10	One(GMT + 5:30)

The tweets were collected in JSON (Java Script Object Notation) format using tweepy, a Python library for accessing Twitter API. These tweets were then stored in data frame format and were finally downloaded in CSV file format. When tweets are downloaded, there is a lot of information associated with them such as information such as: userID, user screen name, number of followers, following date, time, text part of the tweet, device from which tweet has been posted such as android or iOS, location coordinates, user bio, user profile location, user mentions and retweets count. Out of these features, the user screen name, tweet text and user profile location have been selected to predict geolocation of  a tweet. Once the tweets were collected, NLTK^2^ with pip package manager in Python has been used for processing the text in tweets. This process includes the removal of extra places, stop words, URL, emojis, tokenization and lemmatization [15].

**Fig. 1:**
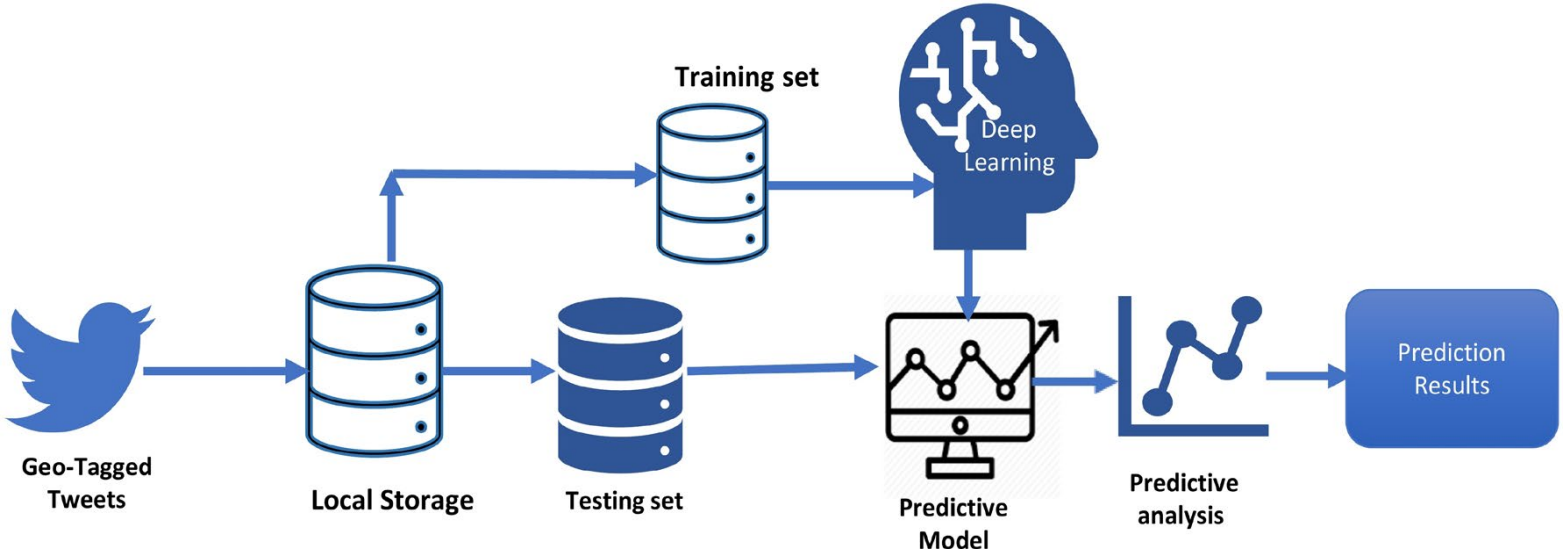
Architecture of the proposed approach

The experiments were performed and results were visualized using Python programming and Keras library with Tensorflow backend. The simulations were performed on the Intel® Core™ i5-8250U CPU @1.80GHz and 64-bit operating system. The framework of the proposed research is shown in Fig. 1.

## Prediction Model

**Fig. 2:**
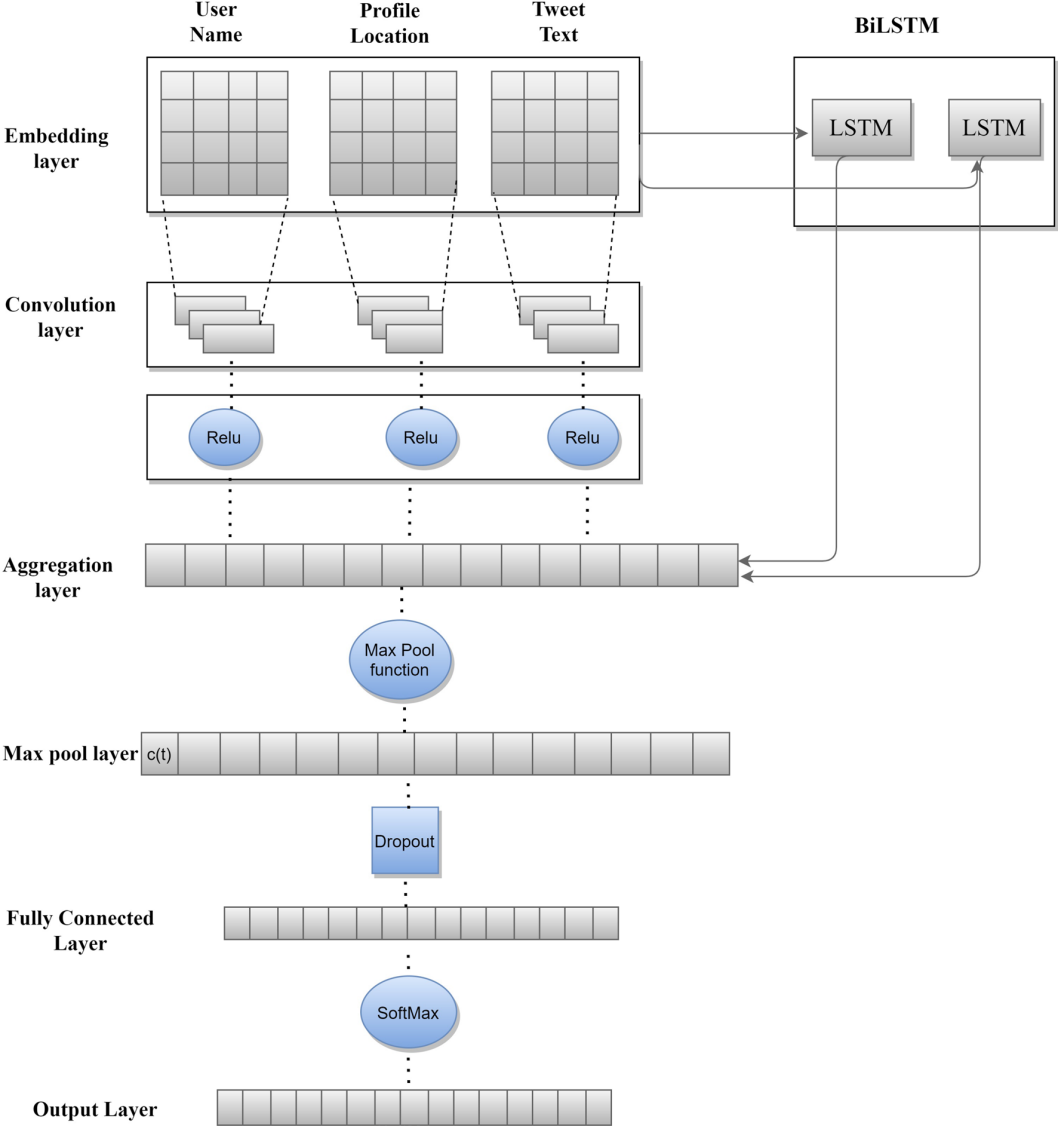
Architecture of the proposed approach

To extract location-specific features from the tweet and its associated attributes, we have used a combination of CNN and BiLSTM as the former has the ability to capture local features and the latter can extract global features from the text. So, location-specific features can be extracted easily by aggregating these two deep learning techniques. The screen name, tweet text and user profile location are the three attributes that have been used to perform the prediction task. We have trained our model using Stochastic Gradient descent with RMSprop with learning rate of 10^-4^. The dataset has been divided in the ratio of 80 by 20; former for training the model and latter for testing the performance of the classifier. The loss function used is sparse categorical cross-entropy. To test the efficiency of our model, we used a fivefold cross-validation technique on our data set. The architecture of our proposed approach is shown in Fig. 2.

Firstly, three text features extracted from the Tweets are concatenated in to a text of length n and then converted in to vector form using word2vec vectors trained on Google GloVe.^3^ Google Glove is an unsupervised algorithm used for obtaining vector representations for words, *W*={*w*_1_, *w*_2_*…w*_n_}*.* The input to our prediction model is word vector obtained from word2vec. These vectors are embedded in embedding layer in form of word matrices *C*_e_. The output of the embedded layer is the tensor reshaped to [512 × 30 ×128 ×1] so that each element of the word vector is itself a list of size 1, instead of a real number. The output of embedded layer is fed to BiLSTM cell as well as convolutional layer simultaneously.
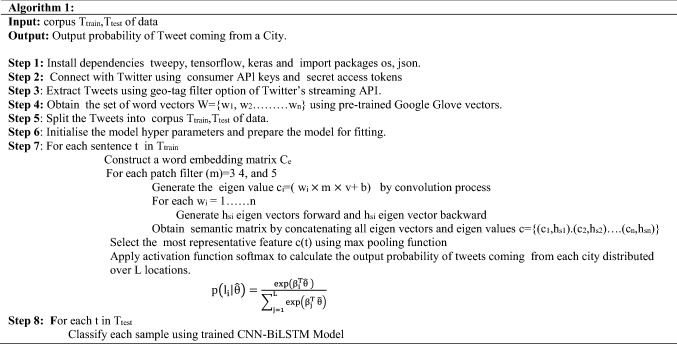


During convolution process, we apply each of 128 filters to all word vector matrices with filter size(*m*) = 3, 4 and 5 with 128 feature vector. The output shape of filter 3,4,5 when applied to a each batch becomes, filter(3) = [512 × 4 × 1 × 128], filter(4) = [512 × 3 × 1 × 128], filter(5) = [512 × 2 × 1 × 128]. Then, we add a bias of 0.1 to the output of convolution layer for convolution of each patch-filter. Since there are 128 filters 128 bias values are used. ReLU is then applied which is a nonlinear function(*x*) = max(*x*,0) where x is the output for each filter size. Table 3 lists the model hyperparameters.

**Table 3:** Model hyperparameters

Batch size	512
Sequence length	30
Number of classes	10
Vocabulary (*v*)	175,409
Embedded vector (*e*)	128
Shape of the Tensor (batch size, sequence length, embedded vector length)	[512 × 30 × 128]
Embedded matrix (*v* × *e*)	175,409 × 128
Epochs	50
Learning rate	10^−4^
Optimizer	Rmsprop

A BiLSTM is a sequence processing model that comprises of two LSTMs: one takes the input in a forward direction, and the other takes it in a backward direction [16]. BiLSTM efficiently increases the amount of information available to the network and improves the context available for the algorithm. BiLSTM cell retains the chronological order between the data by sensing the links between the previous inputs and the outputs. For each step from *i*….*n*, while traversing, a forward LSTM accepts the word embedding of word *w*_i_ and preceding state as inputs, and generates the current hidden state. Similarly, a backward LSTM, on the other hand, reads the text from *w*_n_ to *w*_i_ and generates additional state sequence. The hidden state *h*_*si*_ for word *w*_*i*_ is the combination of *h*_si_ eigen vector forward and *h*_si_ eigen vector backward. Putting together all the hidden states, we get a semantic matrix with location specific features as BiLSTM has provides additional training by traversing the input data twice from left to right and, right to left thus, extracting the semantics of a word in context of the information preceding and succeeding it. The output of convolutional layer, eigen values *c*_i_ = (*w*_i_ × *m* × *v* + *b)* and output of BiLSTM layer, *h*_s_ = {*h*_s1_, *h*_s2_…*h*_sn_} is then combined to generate a sequence, {(*c*_1_, *h*_s1_).(*c*_2_,*h*_s2_)…(*c*_n_, *h*_sn_)*.* In pooling layer max function is applied over the combined output of CNN and BiLSTM to generate maximum value as most representative feature *c*(*t*). Features are then generated in form of vector *θ*. Max pool function also supresses noisy activations along with dimensionality reduction.

A dropout of 0.4 is applied to the output of max pooling layer to prevent the model from overfitting and co-adaptation of hidden units. We add two more features posting time and time zone with one-hot encoding at the end of *θ* and get $\hat{\theta }$. An activation function, SoftMax given in Eq. 1 is then applied to generate the probability of a tweet coming from location li.

1 $$p\left( {\left. {l_{i} } \right|\hat{\theta }} \right) = \frac{{\exp \left( {\beta _{i}^{T} \hat{\theta }~} \right)}}{{\mathop \sum \nolimits_{{j = 1}}^{L} \exp \left( {\beta _{j}^{T} ~\hat{\theta }} \right)}}$$

where *L* is the number of cities in the data set and *β*_*i*_ (weight vectors, word vectors, etc.) are parameters in SoftMax layer. The output predicted location is the city with highest probability. Back propagation algorithm is used to adjust model parameters, word vectors and weight vectors. We have applied stochastic gradient descent over mini-batches with Rmsprop optimizer and sparse categorical cross entropy loss as objective function for classification. This Prediction model can also work for other social networking sites such as the location of Facebook status updated by the users.

## Time and Space Complexity Analysis

The time complexity governs the amount of time an algorithm takes to train and test the model. The time taken by a convolutional neural network to converge is *O*(*m*^2^ *k*^2^ *c*_in_ *c*_out_), where *m* is the size of the output graphs, *k* is the size of the kernel, *c*_in_ is number of units in input layer and *c*_out_ is number of units in output layer. Time taken by a BiLSTM cell is *O*(*m*^2^ *k*^2^ 2*c*_in_ 2*c*_out_) since the input text is traversed twice by forward and backward LSTM cells. Therefore, the algorithm has high computational complexity but effective in terms of space complexity as it gets highly reduced as CNN captures only the high level features from the text and ignores the redundant features while BiLSTM captures global features from the text thereby reducing the size and dimensionality of the feature vector. Further, drop out is applied which drops the trainable parameters in each of the iteration thereby reducing the number of parameters and stopping the model from over-fitting.

## Evaluation Metrics

We have evaluated the performance of our model on different metrics as shown in Table 4.

- *Accuracy* The percentage of correct predicted city locations by total Predictions
- *Acc@top5* The percentage of top five correct predicted city locations.
- *Median* The Euclidean distance between pair of predicted coordinates (*y*’^lat^,*y*’^lon^) and coordinates (*y*^lat^,*y*^lon^) of a city.
  $${\text{Median}} = \sqrt {\left( {y^{{{\prime }lat}} - y^{{lat}} } \right)^{2} - \left( {y^{{{\prime }lon}} - y^{{lon}} } \right)^{2} }$$
  $${\text{Precision}} = \frac{{{\text{True}}\;{\text{Positive}}}}{{{\text{True}}\;{\text{Positive}} + {\text{False}}\;{\text{Positive}}}}$$
  $${\text{Recall}} = \frac{{{\text{True}}\;{\text{Positive}}}}{{{\text{True}}\;{\text{Positive}} + {\text{False}}\;{\text{Negative}}}}$$
  $$F1{\text{ - Score}} = ~\frac{{2 \times {\text{Precision}} \times {\text{Recall}}}}{{\left( {{\text{Precision}} + {\text{Recall}}} \right)}}$$

**Table 4:** Performance of the model

City	Precision	Recall	F1-score	Accuracy	Output probability
Lucknow	0.726891	0.667954	0.696177	0.966944	0.625
Patna	0.834008	0.67541	0.746377	0.969352	0.676
Bhopal	0.90201	0.518038	0.658112	0.959173	0.518
Ahmedabad	0.714286	0.431034	0.537634	0.938813	0.431
Hyderabad	0.566929	0.251309	0.348247	0.941003	0.252
Chandigarh	0.583643	0.291822	0.389095	0.946038	0.290
Bengaluru	0.882102	0.61791	0.726741	0.948884	0.617
Gurugram	0.533279	0.738202	0.619227	0.911559	0.741
New Delhi	0.384338	0.645015	0.481669	0.798818	0.647
Mumbai	0.718521	0.850889	0.779123	0.884194	0.851

## Results and conclusion

**Fig. 3:**
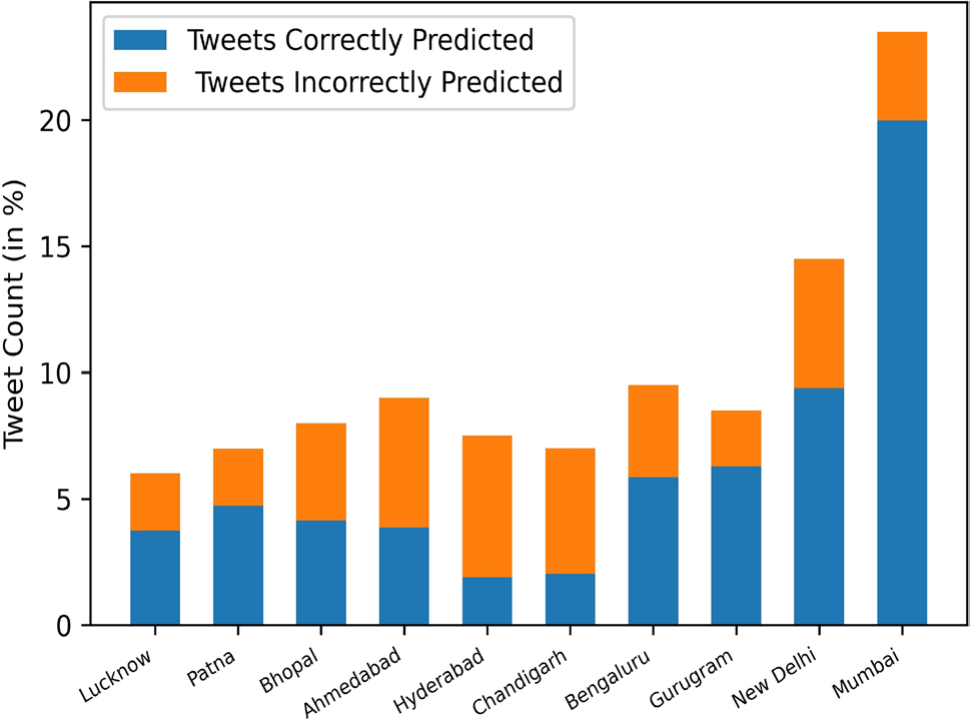
City level prediction results. The height of the blue bar shows percentage of Tweets whose location is predicted correctly from each city. The height of the orange bar shows the percentage of tweets whose location is incorrectly predicted from each city

**Fig. 4:**
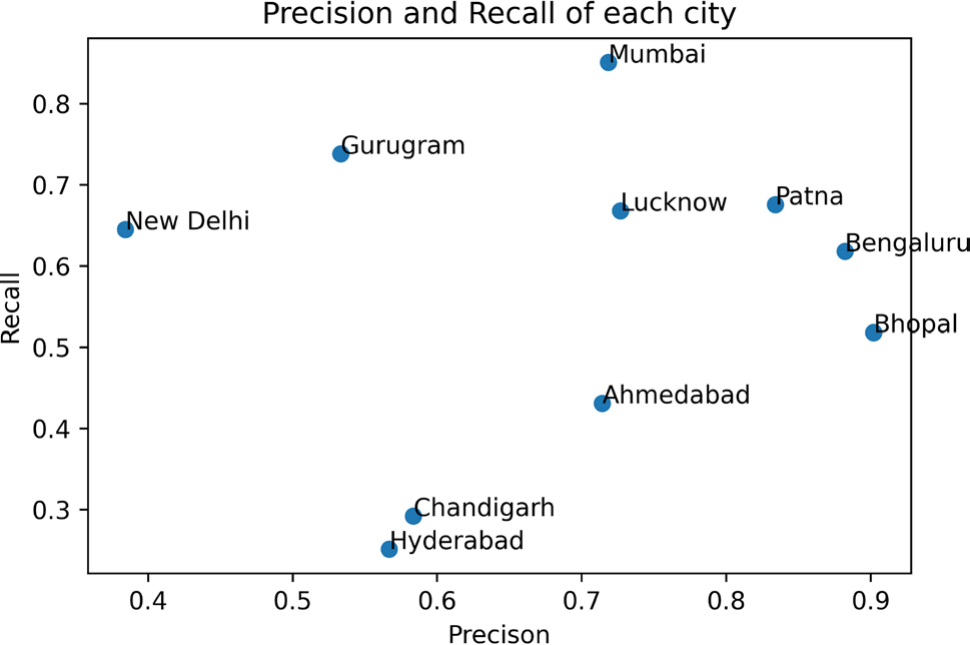
Precision and recall of each city

**Fig. 5:**
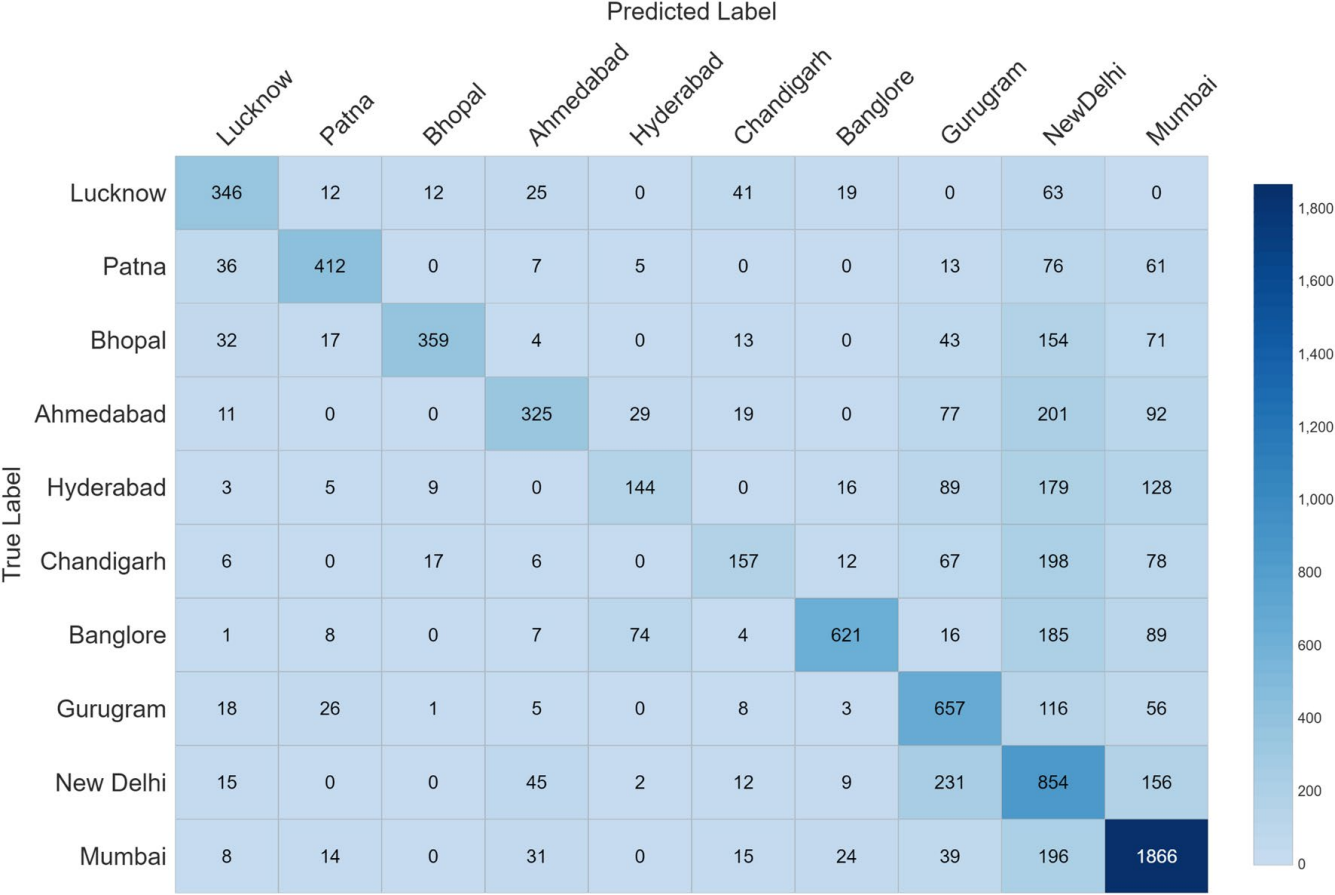
Confusion matrix showing true labels and predicted label

In this paper, we have proposed a deep learning model by combining Convolutional Neural Network (CNN) and a Bidirectional Long Short-term Memory (BiLSTM) to address the problem of geolocation prediction of tweets by extracting features within the tweets and the features associated with the tweets. The job of location prediction of a tweet can be approached as a classification problem, where the aim is to predict city labels for a single tweet or as a multi-variable or a multioutput regression problem, where the goal is to predict latitude and longitude coordinates for a certain tweet. We concentrated on both the approaches in which we first predicted city labels and then extracted longitude and latitude information from labels in order to determine the median error between predicted and true coordinates. Precision, Recall and F1-score has been used to evaluate the performance of our classifier by plotting the confusion matrix. We have also compared our results with previous baseline models and the outcome of our experiment shows a significant improvement over baselines methods achieving an accuracy of 92.6 at the city level prediction with a median error of 22.4 km after evaluating it on fivefold cross validation technique. The comparison results of our approach with previously baseline approaches are listed in Table 5. The graph in Fig. 3 shows the city level prediction result with output probability, Fig. 4 shows precision and recall of each city visually and Fig. 5 shows the confusion matrix. Despite the satisfactory performance of our proposed algorithm, it has high computational complexity. Another limitation of our work was the lack of geo-tagged tweets as most of the Twitter users choose to conceal their geo-location in order to maintain privacy or prevent bullying, stalking or trolling. All the data used in the study is available on Twitter to support further experimentation and analysis. As for the future work, we plan to add open street mapping from Google to capture dynamic movement of the user and images posted by users on the Twitter timeline to our data set.

**Table 5:** Comparison of our approach with previous baselines models for city level prediction

	Accuracy	Acc@Top5	Median (kms)
Han et al. [9]	0.260	–	260
Han et al. [ 10]	0.389	0.595	77.5
Huang and Carley [11]	0.528	0.711	28.0
Huang and Carley [ 13]	0.720	–	28.2
Proposed approach	0.926	0.951	22.4

## Data Availability

All the data used in the study is extracted online from Twitter.

## Footnotes

1 https://developer.twitter.com/en/docs/twitter-api/v1/geo/places-near-location/api-reference/get-geo-reverse_geocode.

2 https://www.nltk.org/.

3 https://nlp.stanford.edu/projects/glove/.
